# The importance of non-fatal overdose in reducing drug related deaths characteristics of non-fatal overdoses and associated risk factors in patients attending a specialist community-based substance misuse service

**DOI:** 10.1177/20494637221115985

**Published:** 2022-08-16

**Authors:** Catriona Matheson

**Affiliations:** Faculty of Social Sciences, 150749 University of Stirling, Stirling, UK

**Keywords:** Overdose, pain medication, drug related death, care pathways, ambulance service

## Abstract

This commentary piece reviews the paper by Cowden et al. on Characteristics on Non-fatal overdoses.

## Commentary

The focus on non-fatal overdose (NFO) (or near fatal) by Cowden et al. is welcomed, in their detailed study in Tayside, an area blighted as having the highest level of drug related deaths in Scotland. To prevent Drug Related Death (DRD), we need to focus upstream and NFO are one step upstream as an indicator of risk of future DRD as highlighted in previous international literature.^1^ Indeed this risk indicator was why the Drug Deaths Taskforce in Scotland identified non-fatal overdose pathways as an area for development in their early strategic plan.^2^

In their study of Cowden et al. describe the demographic profile of those in drug treatment experiencing NFO which maps directly onto the profile of those experiencing DRD across Scotland.^3^ Overdoses and deaths are more prevalent in men and in areas of high deprivation. For both overdose risk and DRD, there is considerable overlap in the socioeconomic profile with other conditions such as chronic pain and mental health problems.^4^ The real challenge for society and government health and social policy is how to get further upstream and address the fundamental challenges of social disadvantage and the associated health inequalities.

However, this paper is from a service improvement perspective and highlights high levels of polydrug use and the role of analgesics, gabapentinoids and benzodiazepines in NFO. Benzodiazepines are a long established and still increasing feature of DRD in Scotland^5^ and the increased implication of etizolam in DRD statistics, which is not prescribable in the UK, has been charted in the national data.^3^ There does appear to be a potential correlation between etizolam use, gabapentinoid use and increased DRD from plots of drugs implicated over time in DRD.^3^

Gabapentinoids have been a relatively recent increasing part of the picture in the polydrug combinations. The source of these drugs is less certain than for etizolam but diverted prescription sources seems at least partly responsible given the increasing prescribing patterns of these drugs generally.^6,7^ However, we also know that people who use drugs experience chronic pain from a range of injuries so may be prescribed these themselves. The reported implications of strong and weak prescribed (or over the counter) analgesics in overdose events described in the paper are relatively small in this sample with just 1.26 and 2.15%, respectively, reported. In the neighbouring area of Fife, a quality improvement exercise assessing levels of overdose risk in the prescribed opioid analgesic in the community found a positive significant relationship between level of social deprivation and level of strong opioid prescribing (*p* < 0.001). People prescribed strong opioids tended to be older (mean 59.7 years) and female (8638, 61.4%) and, among a subset of patients, age, gender and opioid drug class were significantly associated with prescribing of High/Very High doses.^4^

Unfortunately, this study relies on self-report of drugs taken, a limitation that the authors recognise. The amnesic effects of benzodiazepines, particularly in high doses and particularly a feature of etizolam, ^8^ make this a significant challenge to recall reliability. The amnesic effects of benzodiazepines are also clinically important and considered to play a part in overdoses as people forget what they have taken and take more. It is also worth noting here, for this audience that benzodiazepines are prescribed for pain when their muscle relaxing properties are required. However this muscle relaxing effect may contribute to overdose through relaxation of muscles in the neck and chest, potentially exacerbating respiratory depression cause by opiates.

A novel aspect of the paper is the use of Ambulance service data which is an untapped resource that could be used more pro-actively for prevention of harm. In Australia the potential of using ambulance data has also been explored as a means of potential hotspots generated in the heroin market.^9^ As former Chair of the Drug Death Taskforce in Scotland, I was very impressed by the quality and potential of data in the Scottish Ambulance Service as well as the positive attitude of the Scottish Ambulance Service to contribute to addressing drug harm. Real time linking of data into service delivery, ideally using digital means is a way forward to rapidly identify overdose hotspots as well as ‘at risk’ individuals. This will require digital systems to be linked and, ideally Ambulance technicians to have access to rapid oral testing kits to allow them to assess risk through polydrug use, onsite. This is particularly pertinent for those individuals not wanting to be taken to hospital. A taskforce funded research project which has interviewed Ambulance service staff found resistance by some NFO patients to be taken to hospital a source of frustration and wasted time for Ambulance technicians.^10^ This time could be better used for further risk assessment via on site toxicology testing.

There are a couple of other points to highlight in the paper. The location of NFO was interesting. Ambulances attended 45% of cases in public places. The authors note the increased likelihood of a passer-by intervening compared to overdoses occurring at home. This reinforces the need for strong harm reduction messages about not using drugs alone. This risk extends to the chronic pain population who live alone and take the same range of opiates, gabapentinoids and potentially benzodiazepines, albeit from a prescribed source.

For the pain audience, the importance of developing naloxone interventions for those on strong analgesics, especially if combined with other sedative or muscle relaxing medication. This has been trialled in Australia ^11^ and very recently a successful pilot study in Grampian in Scotland – next door to Tayside, proved the feasibility of a naloxone supply and training for risk reduction in chronic non cancer pain.^12^

In conclusion, this paper by et al. raises several interesting points for further development. The focus on non-fatal overdose is crucial and we need to consider and reduce risk at every opportunity and across the healthcare system including at point of making prescribing decisions in primary or secondary care, addiction services, pharmacy and the Ambulance services.
